# Development of a novel in vitro assay to evaluate environmental water using an IL-8 reporter cell line

**DOI:** 10.17179/excli2020-2104

**Published:** 2020-07-21

**Authors:** Yutaka Kimura, Chizu Fujimura, Toshifumi Imagawa, Socorro P. Lupisan, Mariko Saito-Obata, Mayuko Saito, Hitoshi Oshitani, Setsuya Aiba

**Affiliations:** 1 Department of Dermatology, Tohoku University Graduate School of Medicine, 1-1 Seiryo-cho, Aoba-ku, Sendai, Miyagi 980-8574, Japan; 2 Department of Virology, Tohoku University Graduate School of Medicine, 2-1 Seiryo-cho, Aoba-ku, Sendai, Miyagi 980-8575, Japan; 3 Research Institute for Tropical Medicine, FCC, Alabang, Muntinlupa 1781, Philippines

**Keywords:** toll-like receptors, NOD-like receptors, IL-8, Luciferase assay, monocyte, hapten

## Abstract

The IL-8 luciferase reporter cell line, THP-G8 cells, used in the *in vitro* sensitization test, OECD442E, can respond to a variety of stimuli other than haptens, such as lipopolysaccharide (LPS), other bacterial toxins, and detergents. Considering these characteristics, we examined the ability of the IL-8 luciferase assay using THP-G8 cells to evaluate water pollution. We first stimulated THP-G8 cell with various Toll-like receptor (TLR) agonists and nucleotide-binding oligomerization domain-like receptor (NLR) agonists, and found that TLR1, 2, 4, 5, 6 agonists and NOD 1, 2 agonists significantly augmented IL-8 luciferase activity (IL8LA). Then, we examined the detection threshold of LPS by THP-G8 cells, and found it 0.4 EU/ml. Next, we examined whether THP-G8 cells can differently respond to a variety of sources of environmental water around Sendai, Japan and Manila, Philippine and whether there is a correlation between the IL8LA of different sources of water and their level of endotoxin assessed by the LAL assay. There was a clear trend that the IL8LA was lower in the upper stream and higher in the downstream in both Japan and Philippine. Moreover, there was a strong correlation between the IL8LA of the environmental water and its endotoxin level. Finally, using N-acetyl-L-cysteine, an antioxidant/radical scavenger, and polymyxin B that neutralizes endotoxin, we demonstrated that there was a difference in the suppressive effects by them between the water from Japan and that from Philippine. These data suggest the potential of the IL-8 luciferase assay for evaluating environmental water pollution both quantitatively and qualitatively.

## Introduction

Environmental quality monitoring of surface waters is fundamental to the sustainable management of water resources and to reducing risks posed by multiple anthropogenic stressors (Geissen et al., 2015[6]). Surface waters typically contain complex mixtures of microorganisms, microbial products like endotoxins (Carlson et al., 2013[1]; da Silva et al., 2013[3]) and micropollutants including pharmaceuticals, pesticides, industrial chemicals and their transformation products (Loos et al., 2009[18]). These compounds are emitted to surface water by point sources including treated or untreated wastewater from municipal and industrial sources or livestock enterprises and by diffuse sources such as run-off from urban and agricultural areas (Heeb et al., 2012[11]). These microbial and chemical mixtures may cause adverse effects to aquatic organisms and may pose a risk to human health.

*In vitro* bioassays are increasingly applied for water quality monitoring to detect the effects of complex chemical mixtures (Schroeder et al., 2016[26]; Tousova et al., 2017[32]). Bioassay test batteries covering different stages of cellular toxicity pathways such as induction of xenobiotic metabolism and receptor-mediated effects, as well as apical effects in whole organisms, have been recommended to ensure that a range of possible effects in water are detected (Neale et al., 2017[21]). 

Lipopolysaccharides (LPSs), which are the outer membranes of gram-negative bacterial or cyanobacterial cells, are also widely known to cause strong innate immune reactions in humans via Toll-like receptors (TLRs) on the cell surface. LPS are also called endotoxins because of their biological activity (reviewed by Mazgaeen and Gurung, 2020[19]). Detection of endotoxin contamination has so far been done by the *in vivo* rabbit pyrogen test, the in vitro Limulus Amoebocyte Lysate (LAL) test and monocyte-activation test (MAT). The rabbit pyrogen test measures the change in body temperature after injection of test substance but has limitations in its utility due to insufficient accuracy and requirement of large number of animals (Grant, 1950[8]; van Dijck and van de Voorde, 1977[33]). In addition, the pyrogen response in a rabbit or arthropod does not necessarily reflect the actual human immune response to pyrogenic challenges (Hartung and Human(e) Pyrogen Test Study Group, 2002[10]; Schindler et al., 2003[25]). On the other hand, LAL test detects only LPS endotoxins from the cell wall components of gram-negative bacteria (Levin and Bang, 1968[17]; Tanaka and Iwanaga, 1993[30]) and does not necessarily correlate with *in vivo* biological activities of endotoxins (Pearson et al., 1982[23]; Takayama et al., 1984[29]). 

On the other hand, in the internationally validated protocol of the MAT, the sample is incubated with fresh or cryopreserved human whole blood, and the proinflammatory cytokine interleukin-1β is detected by enzyme-linked immunosorbent assay. In contrast to the LAL test, it can detect non-lipopolysaccharide toxins, such as lipoteichoic acid, exotoxins and fungal components (Daneshian et al., 2009[4]). Although this assay has the commercially available kit now, however, it needs human blood.

We established an IL-8 reporter cell line, THP-G8, derived from a human monocyte cell line, that harbors stable luciferase orange (SLO) and stable luciferase red (SLR) genes under the control of the IL-8 and G3PDH promoters, respectively (Takahashi et al., 2011[28]). This cell line was established for the purpose of screening the immunotoxicity of various chemicals, such as environmental pollutants, and drugs. Indeed, the usefulness of this cell line has been already demonstrated in hazard identification of skin sensitizing chemicals (Kimura et al., 2015[14], 2018[16]; OECD, 2018[22]; Takahashi et al., 2011[28]), characterizing immunotoxic chemicals (Kimura et al., 2014[13], 2018[15]), and quantifying the immunotoxicity of Asian desert dusts (Watanabe et al., 2014[35], 2015[36]). These studies also ensure the stability of this cell line. Reflecting the property of monocyte or macrophage to produce IL-8 in a variety of stimuli including electrophiles like haptens (Toebak et al., 2006[31]), detergents (Coquette et al., 1999[2]), ROS, several cytokines, and lipopolysaccharide (reviewed by Roebuck, 1999[24]), THP-G8 cells can also increase IL-8 promoter driven SLO luciferase activity by these stimuli. 

In contrast to the MAT that quantitatively measures IL-1β secreted by monocytes, the IL-8 luciferase assay using THP-G8 measures the luciferase activity driven by IL-8 promoter. Wang et al. reported by using cDNA array analysis that, in human monocytes, the highest activated genes for proinflammatory mediators induced by bacterial stimulants (*Staphylococcus aureus*, peptidoglycan, endotoxin) were chemokine genes such as IL-8 and macrophage inflammatory protein (MIP)-1α, which were followed by cytokine genes such as TNF-α, IL-1, and IL-6 (Wang et al., 2000[34]). Therefore, the assay using THP-G8 cells seems to be suitable for detection of bacterial toxins.

In this study, to develop an *in vitro* high throughput assay system that can widely screen water pollution, we first determined to what kinds of TLR agonists and Nucleotide-binding oligomerization domain-like receptor (NLR) agonists stimulate THP-G8 cells to augment IL-8 promoter driving luciferase activity (IL8 LA). Next, we examined the IL8LA of different sources of environmental water around Sendai, Japan and Manila, Philippine and the correlation between the IL8LA and their level of endotoxin. Finally, using N-acetyl-L-cysteine (NAC) and polymyxin B, we tried to characterize the pollutants in the environmental water. We designated this assay system using THP-G8 cells to detect the pollution of environment water as the IL-8 Luc assay (W) considering a minor difference in the protocol from the IL-8 Luc assay to evaluate the sensitizing potential of chemicals.

## Materials and Methods

### Water sampling

Environmental water around Sendai city was collected on April 6^th^ 2014. It was sunny weather and the highest temperature was 10.2°C. The water was collected at i) Okura dam (northern latitude of 38°19’32”, eastern longitude of 140°42’22”), ii) the midstream of Hirose river (northern latitude of 38°15’56”, eastern longitude of 140°51’18”), iii) the downstream of Natori river (northern latitude of 38°11’59”, eastern longitude of 140°55’5”), iv) Teizan canal (northern latitude of 38°8’13”, eastern longitude of 140°55’57”), and v) Iwanuma sea shore (northern latitude of 38°8’3”, eastern longitude of 140°56’35”). Environmental water around Manila city was collected on December 13^th^ 2012 at i) Pasig river No. 6 (Pas R6; northern latitude of 14°35’37”, eastern longitude of 121°5’31”), ii) Pasig river No. 4 (Pas R4; northern latitude of 14°33’25”, eastern longitude of 121°4’12”), iii) Pasig river No. 2 (Pas R2; northern latitude of 14°35’21”, eastern longitude of 121°0’49”), iv) Paranaque river No. 4 (Par R4; northern latitude of 14°30’14”, eastern longitude of 120°59’39”), v) Paranaque river No. 3 (Par R3; northern latitude of 14°29’53”, eastern longitude of 120°59’37”), and vi) Las Pinas river No. 4 (Las R4; northern latitude of 14°28’30”, eastern longitude of 120°58’33”). The water was collected using 50 mL conical centrifuge tube (Falcon, Corning, NY) and stocked in a -80 °C freezer.

### Luciferase assay

We used THP-G8 cells derived from a human acute monocytic leukemia cell line THP-1 cell line, which contained stable luciferase orange gene (SLO) regulated by IL-8 promoter and stable luciferase red gene (SLR) by G3PDH promoter (Kimura et al., 2015[14], 2018[16]; OECD, 2018[22]; Takahashi et al., 2011[28]). THP-G8 cells (5x10^4^ cells/100 µL/well) in 96-well black plates (Greiner bio-one GmbH, Frickenhausen, Germany) were cultured for 6 hours with lipopolysaccharide (LPS, from *E. coli *026:B6 (> 10,000 EU/mg, Sigma, St. Louis, MO), TLR agonists (InvivoGen, San Diego, CA), NLR agonists (InvivoGen, San Diego, CA), representative haptens, methylisothiazolinone and glutaraldehyde or 10 µL of the collected environmental water autoclaved for 20 min at 121°C and 2 atm. In some experiments, the cells were pretreated with inhibitors such as N-acetyl-L-cysteine (NAC, Sigma, St. Louis, MO) or polymyxin B (Sigma, St. Louis, MO) for 30 min. The luciferase activities of THP-G8 cells, IL-8 driven SLO-luciferase activity (IL8LA) and GAPDH driven SLR-luciferase activity (GAPLA), were detected by a microplate-type luminometer with a multi-color detection system (Phelios; Atto Co., Tokyo, Japan) using Tripluc^TM^ luciferase assay reagent (TOYOBO Co., Ltd., Osaka, Japan). Since some chemicals affected cell viability, we used the normalized IL8LA (nIL8LA) calculated by dividing IL8LA by GAPLA, and calculated the induction of IL8LA (Ind-IL8LA) and the %suppression as follows.

Ind-IL8LA = nIL8LA treated with toxins or environmental water/nIL8LA without treatment

%suppression = (1-Ind-IL8LA of THP-G8 cells pretreated with the inhibitor/Ind-IL8LA) x 100.

### Limulus amebocyte lysate (LAL) assay

The collected environmental water was autoclaved for 20 min at 121 °C and 2 atm, and diluted 1000 times with endotoxin free water. Endotoxin level was measured using Toxin Sensor^TM^ Chromogenic LAL EndotoxinAssay Kit (GeneScript, Piscataway, NJ) according to the manufacturer’s instructions. To determine the activity of LPS from *E. coli* 026:B6, 1 mg/ml of LPS solution was serially diluted and the diluted samples were examined by both IL-8 Luc assay (W) and THP-G8 cells and the LAL assay. Then, using the calibration curve obtained by the Endotoxin Assay Kit, we determined the endotoxin activity of LPS from *E. coli* 026:B6.

### Statistical analysis

The statistically significant induction of Ind-IL8LA by LPS was determined by t-test. *p* values ≤0.05 were considered statistically significant. Correlation between endotoxin level (EU/mL) and IL8LA was assessed by Pearson’s correlation coefficient analysis. 

## Results

### Response of THP-G8 cells against agonists of TLRs and NLRs

**Figure 1:**
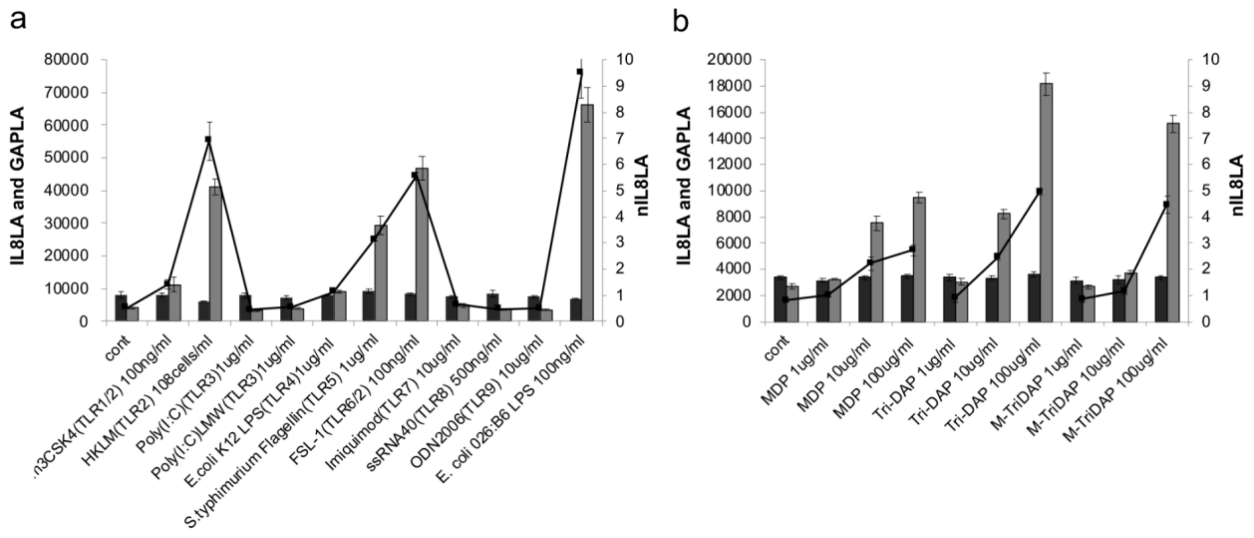
Response of THP-G8 cells against agonists of TLRs and NLRs. THP-G8 cell (5x10^4^ cells/100 µL/well) in 96-well plate were stimulated with agonists of TLRs (a) and NLRs (b). After cells were incubated 6 hours at 37 °C with 5 % CO_2_, the luciferase activity was measured. The data represented means ± SD (n=4) and one representative data from two independent experiments were shown. Dark gray bar: GAPLA, Light gray bar: IL8LA, Line: nIL8LA.

We first examined which TLR or NLR agonists could stimulate nIL8 LA of THP-G8 cells. When THP-G8 cell in 96-well plate were stimulated with agonists of a series of TLRs and NLRs for 6 h, they increased IL8LA by the stimulation with TLR 1, 2, 4, 5, and 6, and NLR 1 and 2 agonists, while GAPLA that reflected cell survival rate did not significantly change (Figure 1(Fig. 1)). 

### The sensitivity of the response of THP-G8 cells against lipopolysaccharides

**Figure 2:**
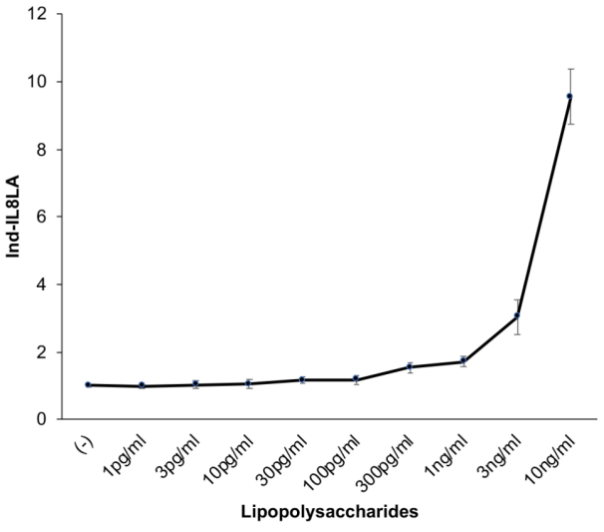
Response and sensitivity of THP-G8 cells against lipopolysaccharides. THP-G8 cell (5x10^4^ cells/100 µL/well) in 96-well plate were stimulated with various concentrations of lipopolysaccharides (*E. coli* 026:B6). After cells were incubated for 6 hours at 37 °C with 5 % CO_2_, the luciferase activity was measured. The data represent means ± SD (n=4) and one representative data from two independent experiments was shown.

We next determined the minimum concentration of lipopolysaccharides (*E. coli* 026:B6) to significantly stimulate the IL-8LA of THP-G8 cell. When stimulated with the graded concentrations of lipopolysaccharides, LPS significantly increased IL-8 LA evaluated by Ind-IL8LA in a dose-dependent manner of LPS at the concentration equal to or higher than 0.03 ng/mL, which corresponded to approximately 0.4 EU/ml determined by the LAL assay (Figure 2(Fig. 2)).

### Response of THP-G8 cells against the environmental water

**Figure 3:**
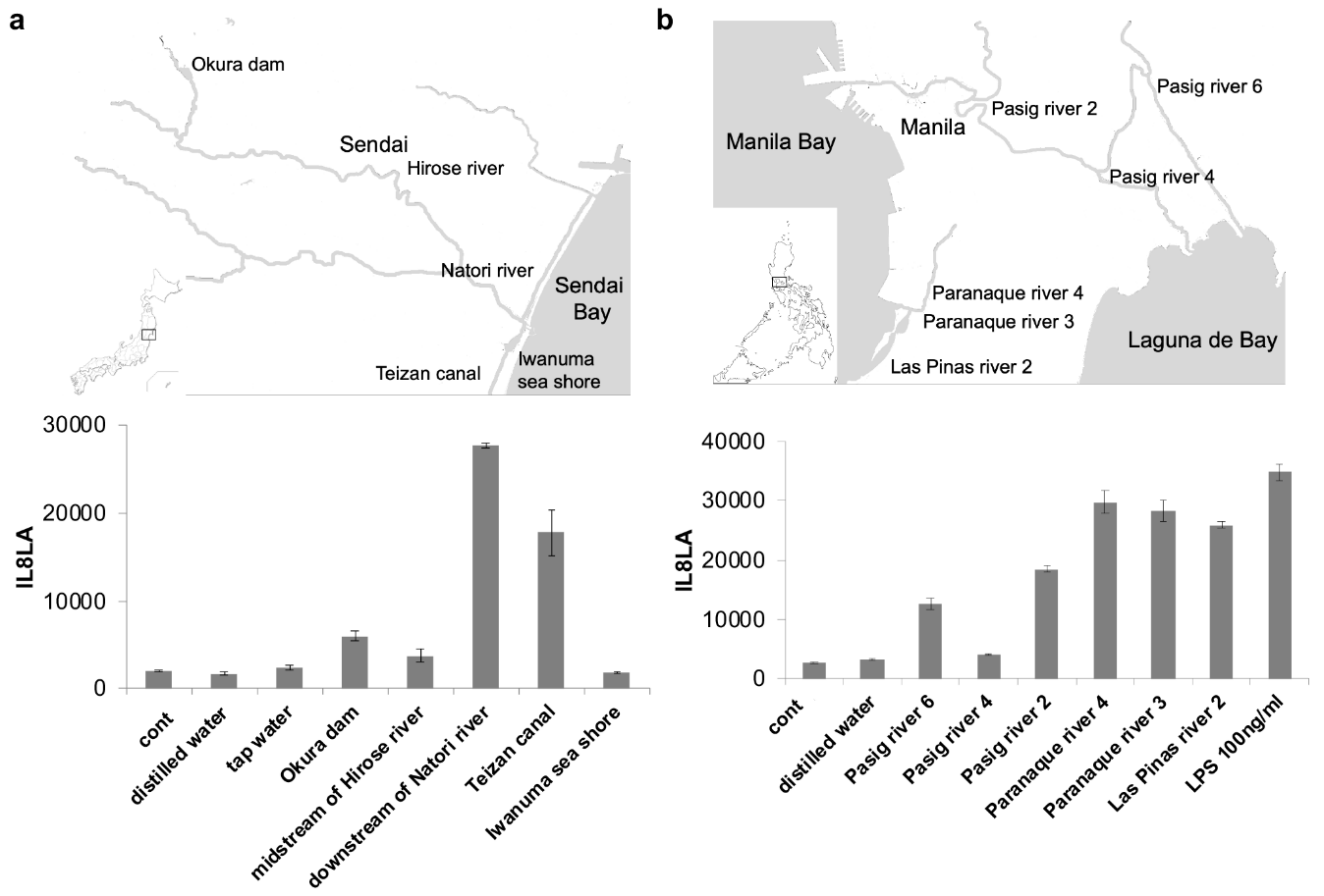
Response of THP-G8 cells against the environmental water. THP-G8 cell (5x10^4^ cells/100 µL/well) in 96-well plate were stimulated with 10 µl of the autoclaved environmental water around Sendai, Japan (A) and Manila, the Philippines (B). After cells were incubated for 6 hours at 37 °C with 5 % CO_2_, the luciferase activity was measured. The data represented means ± SD (n=4) and one representative data from two independent experiments was shown.

Next, we examined the IL8LA of the environmental water at various spots along the rivers around Sendai, Japan and Manila, the Philippines. Although the environmental water from all the spots increased IL8LA at various levels, in general, the level of the IL8LA was lower in the upper stream and higher in the downstream. Namely, IL8LA was slightly augmented by the water from the Okura dam located at the upper stream of Hirose river and from the midstream of Hirose river. On the other hand, it was strongly augmented by the water from the downstream of Natori river and Teizan canal close to the outlet of the river (Figure 3a(Fig. 3)). Similarly, in Manila, IL8LA were highly augmented by the water from the downstream, Paranaque river 4, Paranaque river 3, Las Pinas river 2, compared with water from the upper stream, Pasig river 4, Pasig river 6, Pasig river 2 (Figure 3b(Fig. 3)). Seawater did not augment IL8LA. The water of the downstream of both Sendai and Manila stimulated IL8LA with a magnitude similar to LPS 100 ng/mL.

### Correlation of IL8LA measured by THP-G8 and LAL assay result of the environmental water around Sendai and Manila

**Figure 4:**
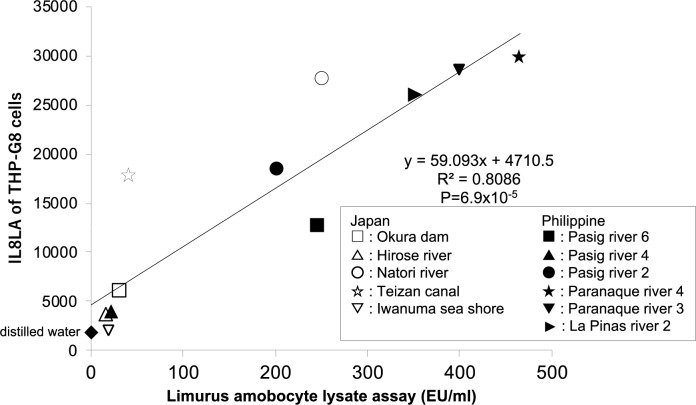
Correlation between IL8LA and endotoxin level in the environmental water around Sendai and Manila. The IL8LA and the endotoxin level of a various sources of environmental water from Sendai and Manila was examined by the IL-8 Luc assay (W) and the LAL assay.

We compared the correlation between the endotoxin activity measured by LAL assay and the activity to stimulate IL8LA of the environmental water. There was a significant correlation between them (R2 = 0.8086, *p* = 0.000069). However, some samples such as the water from Natori river and Teizan canal showed IL8LA higher than the IL8LA estimated by the endotoxin level, which suggests that this water contained the substance other than LPS that could stimulate THP-G8 cells (Figure 4(Fig. 4)). 

### Suppression of IL8LA induced by the environmental water around Sendai and Manila by polymyxin B and N-acetyl cysteine

Since THP-G8 cells can respond to a variety of stimuli including electrophiles like haptens, detergents, ROS, several cytokines, and lipopolysaccharide, the environmental water may stimulate THP-G8 cells by xenobiotics and toxins other than LPS. Indeed, the discrepancy between the IL8LA and the endotoxin level of Natori river and Teizan canal suggest the contamination of some substances other than LPS. It is well known that polymyxin B neutralizes the lethal toxicity, limulus gelation activity, and hemodynamic effects of the endotoxin by binding to the lipid A domain, which is the active center of the endotoxin molecule (Morrison and Jacobs, 1976[20]). On the other hand, NAC functions by its antioxidant/radical scavenger properties or its thiol-disulfide exchange activity as a reductant (Kim et al., 2001[12]). Therefore, it is conceivable that the pretreatment with these chemicals can characterize the contaminants in the environmental water that stimulates IL8LA. 

**Figure 5:**
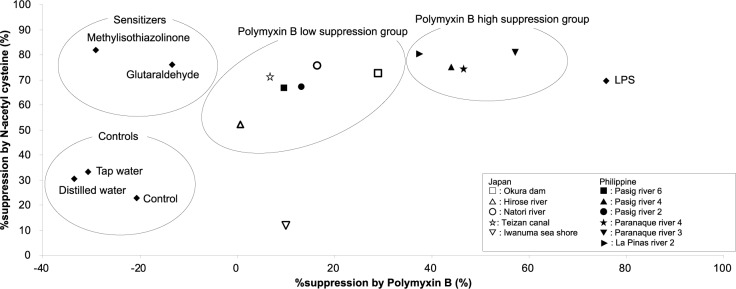
Suppression of IL8LA induced by the environmental water around Sendai and Manila by polymyxin B and N-acetyl cysteine (NAC). The IL8LA of the environmental water around Sendai (square) and Manila (triangle), skin sensitizers (2.5 µg/mL of methylisothiazolinone, 5 µg/mL of glutaraldehyde), and 100 ng/mL of LPS was measured with or without the pretreatment of 50 mg/mL of polymyxin B or 25 mM of NAC for 30 min. The %suppression by polymyxin B and NAC was plotted on X axis and Y axis, respectively. Minas value of %suppression by polymyxin B means that the pretreatment of polymyxin B increased Ind-IL8LA.

We first examined the effects of these inhibitors on IL8LA stimulated with LPS or haptens. The %suppression of polymyxin B and NAC on IL8LA stimulated by LPS was 75.8 and 69.8, respectively. On the other hand, IL8LA stimulated by haptens, methylisothiazolinone and glutaraldehyde, were suppressed by pretreatment of NAC (%suppression=82.0 and 76.2, respectively), but not suppressed by polymyxin B (%suppression=-29.1 and -13.4, respectively). Then we examined their effects on IL8LA stimulated with the environmental water around Sendai and Manila, and the %suppression by polymyxin B and NAC was plotted on X axis and Y axis, respectively (Figure 5(Fig. 5)). This graph indicated that the environmental water could be divided into two groups, the polymyxin B high suppression group that mostly consists of the environmental water around Manila and the polymyxin B low suppression group that mainly consists of the environmental water around Sendai. The %suppression by NAC was slightly lower in the polymyxin B low suppression group. These data suggest that THP-G8 cells enable us to evaluate the environmental water qualitatively and quantitatively, using inhibitors in combination.

## Discussion

In this study, we first demonstrated that TLR 1, 2, 4, 5, 6, NLR 1 and 2 agonists augmented IL8LA and that LPS dose-dependently stimulated IL8LA with the lowest concentration of 0.03 ng/ml or 0.4 EU/ml. In addition, we have already reported that most of haptens as well as detergents also stimulated IL8LA (Kimura et al., 2015[14], 2018[16]). Therefore, IL-8 Luc assay (H) can respond to a variety of environmental contaminants other than endotoxin, such as bacterial toxins from gram positive and negative bacteria and chemical pollutants such as haptens and detergents. It is one of the advantages of this assay over the LAL assay.

THP-G8 can detect LPS at the concentration of higher than 0.03 ng/ml or 0.4 EU/ml (Figure 2(Fig. 2)). This concentration was much lower than the concentration of the lowest limit of LPS (2 ng/ml) to stimulate IL-8 production by THP-1 cells, which was reported by Schwarz et al. (2014[27]). Since 0.5 EU/ml is widely accepted as the fever threshold (Gorbet and Sefton, 2005[7]; Takayama et al., 1984[29]), the sensitivity of the IL-8 Luc assay (W) might be sensitive enough for pyrogen test. 

Recently, there are urgent needs for *in vitro* bioassays which can assess endotoxin levels which affect human immune system more sensitively and in a more high-throughput way. So far, the endotoxin levels of the environment were estimated by LAL assay. However, there are several reports that there is a poor correlation between the potency of endotoxin determined by the LAL assay and the assays determined by using human cells (Dehus et al., 2006[5]; Hansen et al., 1999[9]). Considering that IL-8 is one of the highest activated genes in human monocytes induced by bacterial stimulants (Wang et al., 2000[34]), the reporter cell line using the IL-8 promoter is reasonable. So, this is the second advantage of this assay over the LAL assay. 

In contrast to the LAL assay, the IL-8 Luc assay (W) lacks the specificity because it respond to toxins other than LPS and some chemicals. To overcome this problem, we pretreated THP-G8 cells with polymyxin B and NAC. Using these compounds, the IL-8 Luc assay (W) clearly discriminated the increased IL8LA by LPS from that by haptens. Namely, the former was significantly suppressed by both polymyxin B and NAC, while the latter was significantly suppressed only by NAC. Similarly, the environmental water we examined was classified into two group, the polymyxin B high suppression group that mostly consists of the environmental water around Manila and the polymyxin B low suppression group that mainly consists of the environmental water around Sendai. This results suggests that the increased IL8LA by the water from Manila is mostly caused by the contamination of LPS, while that from Japan is at least partially by the contamination of chemicals or toxins other than LPS. This result is just a representative. If the IL-8 Luc assay (W) is combined with other inhibitors, it becomes possible to identify the contaminants that increase IL8LA. This is the third advantage of the IL-8 Luc assay (W) over the LAL assay.

Finally, the procedure of this assay is simple and reproducible. Namely, the culture of THP-G8 cells is relatively easy and does not require the use of trypsin or EDTA. In this assay, the environmental water or chemicals at graded concentrations are added to the wells of a 96-well culture plate. Then, cells adjusted to the optimum concentration are seeded into each well. After 6 h incubation, 100 μl of pre-warmed Tripluc is added to each of the 96 wells. The subsequent process is completely automated. Therefore, the IL-2 Luc assay is a test method that can significantly reduce human error. Moreover, the procedure of the IL-8 Luc assay (W) was almost identical with that of the OECD442E. In other words, the procedure is considered to have been officially validated. This is the fourth advantage of this assay.

Finally, considering the advantage of the IL-8 Luc assay (W) over the gold standard of LAL assay, the IL-8 Luc assay (W) can be used for evaluating the environmental water, even for regulatory purpose. 

## Acknowledgements

This work was supported by JSPS KAKENHI (Grant-in-Aid for challenging Exploratory Research, 25550076), Grants-in-Aid from the Ministry of Health, Labor and Welfare (MHLW, 0618010), and Japan Initiative for Global Research Network from Japan Agency for Medical Research and Development (JP19fm010813 and JPwm0125001).

## Conflict of interest

The authors have no conflict of interest to report.
